# Australian Notes

**Published:** 1918-09-21

**Authors:** 


### AUSTRALIAN NOTES.

### Admissions to Hospitals.

SYDNEY'S Hospital Admission Depôt has set Melbourne thinking, and official attention at present is bent upon smoothing the way of the sick to the most suitable institutions for their ailments. Dr. Robertson, Chief Health Officer, states that "a goodly number of sick persons come to the Health Department for treatment thinking that the department controls hospitals generally," and the Minister for Health—Mr. Bowser— is to bring the matter before the Cabinet.

### Doctors and Lodges: The Commission's Finding.

AT a special meeting on June 26 the committee of the Victoria branch of the B. M. A., by a unanimous vote, and the next day the Friendly Societies' Association "almost unanimously," decided to accept Judge Wasley's finding, which was published in THE HOSPITAL of September 14, page 518. Since the "Doctors *versus* Lodges" dispute began, nine new medical institutions have been formed. It is understood that the Friendly Societies are extremely reluctant to suppress these organisations, which have cost much money and thought and are giving satisfaction.

### Personal.

DR. R. P. MCMEEKIN, medical superintendent of the Melbourne Hospital since 1914, was reappointed for another year on June 18. The president, Sir John Grice, on behalf of the committee, congratulated Dr. McMeekin on the excellent services he had rendered.

